# Estimated incidence rate and distribution of tumours in 4,653 cases of archival submissions derived from the Dutch golden retriever population

**DOI:** 10.1186/1746-6148-10-34

**Published:** 2014-01-31

**Authors:** Kim M Boerkamp, Erik Teske, Lonneke R Boon, Guy CM Grinwis, Lindsay van den Bossche, Gerard R Rutteman

**Affiliations:** 1 Faculty of Veterinary Medicine, Department of Clinical Sciences Companion Animals, Utrecht University, Yalelaan 108, Utrecht 3584 CM, The Netherlands; 2 Faculty of Veterinary Medicine, Department of Pathobiology, Utrecht University, Yalelaan 1, Utrecht 3508 TD, The Netherlands

## Abstract

**Background:**

A genetic predisposition for certain tumour types has been proven for some dog breeds. Some studies have suggested that this may also be true for the Golden retriever breed. The present study aimed to examine a possible existence of a tumour (type) predisposition in the Dutch population of Golden retrievers by evaluating annual estimated incidence rates compared to incidence rates from previous publications. A second aim was to evaluate whether incidences of various tumours differed as related to the diagnostic method chosen, being either cytology or histology.

**Results:**

Tumours submitted to Utrecht University during the period 1998–2004 diagnosed either by means of cytology (n = 2,529) or histology (n = 2,124), were related to an average annual Dutch kennel club population of 29,304 Golden retrievers.

Combining individual tumours from both the cytological and the histopathological data-set resulted in an annual estimated incidence rate of 2,242 for 100,000 dog-years at risk regarding tumour development in general.

The most common cytological tumor diagnoses were ‘fat, possibly lipoma’ (35%), mast cell tumour (21%) and non-Hodgkin lymphoma (10%). The most commonly diagnosed tumours by histology were mast cell tumour (26%), soft tissue sarcomas (11%) and melanoma (8%). Both the cytological and histopathological data-sets, showed variation; in patient age distribution, age of onset and incidence of various tumours.

**Conclusion:**

Comparing our data with previous reports in non-breed-specified dog populations, the Golden retriever breed shows an increased risk for the development of tumours in general, as well as an increased risk for the development of specific tumour types, including the group of soft tissue sarcomas. Variations in age, location and incidence of various tumours were observed between the two data-sets, indicating a selection bias for diagnostic procedure.

## Background

Breeding from within a selected population of dogs can, in a relative short period of time, give rise to a clear change in phenotype which leads to breed development [1,2] but may also cause an increase in the occurrence of inherited diseases [3-7] such as cancer [8-12]. Clear evidence exists for a breed-related predisposition to specific cancers, like histiocytic sarcomas in Bernese Mountain dogs [10,13] and Flatcoated retrievers, [11] anal sac carcinomas in the English Cocker Spaniel [12,14] and hemangiosarcomas in German Shepherd dogs [15]. For the Golden retriever, an increased risk for the development of cancer in general has been reported by some [16,17], but not all studies [18,19]. Also, there are reports on an increased risk for specific types of cancer in Golden retrievers such as mast cell tumours (MCT) [12,20] melanomas [21] and non-Hodgkin lymphomas (NHL) [12,22,23] and suggestions of a predisposition to soft tissue sarcomas (STS) [24-27]. The limitations of these studies are old age in the dogs, and the fact that many studies were hospital-based and of small size. Larger studies often rely on data from insurance companies without verification of cytological or histopathological diagnoses. There is need for larger studies that include examination of reports of diagnostic procedures to obtain more solid data on the relative tumour incidence. Such larger studies should enable better assessment of a possible predisposition for specific tumour types in breeds such as the Golden retriever. The results may serve as means to improve health of the breed as well as basis for comparative oncological research [12,16]. Furthermore, regional variation in genetic population structure may appear [28].

Our aim was to obtain an estimate of the occurrence of tumours and the distribution of tumour types in the Golden retriever breed in the Netherlands. This was done by accessing the archives (1998 – 2004) from two of the main laboratories in the Netherlands – both located at the Faculty of Veterinary Medicine in Utrecht - that independently provide histopathological (Veterinary Medical Diagnostic Center) - or cytological diagnostic services (University Veterinary Diagnostic Laboratory).

Diagnostic management of dogs suspected to be affected with neoplasia may preferentially be done by cytological examination of fine needle aspiration biopsies (FNABs), by histopathological examination of resected masses, or by both. Which method is chosen, depends on multiple factors like accessibility of a possible neoplastic mass, the suspected tumour type, financial aspects, the availability of an experienced laboratory, etc. Most studies that investigated the incidence of cancer in dog populations have been based solely on histopathology or are unclear about the diagnostic method used. Our data-sets considered both histopathological- and cytological examination; separately and combined.

This retrospective study can, besides providing information on potential health risks within the Golden retriever, also be of help to veterinarians by providing possible differential diagnoses.

## Results

Of a total of 4,313 fine needle aspiration biopsies (FNAB) of masses taken from Golden retriever dogs, a total of 2,529 cases were diagnosed as a suspected tumour (specific details are listed in Table 1). Remaining biopsies were either non-diagnostic, mostly due to poor cellularity of the specimen (n = 739; 17%) or diagnostic, but not considered to originate from a neoplasm (n = 1,045; 24%). Of these non-neoplastic lesions, 51% were diagnosed as inflammation.

**Table 1:** Patients characteristics

	**Cytological data-set**	**Histological data-set**	**Combined set (excluding 54 double entries)**
Number of tumours	2,529	2,124	4,599
• Mean/year	361	303	657
• Nr of dogs in which a second tumour was detected	69	46	115
• Nr of dogs in which a third tumour was detected	0	24	24
Male/male neutered	795/365	719/320	
Female/female neutered	530/649	413/600	
Median age	9.1 yrs	8.6 yrs	
	(min. 0.2 yrs, max.17.2 yrs)	(min. 0.1 yrs, max.17.2 yrs)	
Number of malignant tumours	1,203 (48%)	1,262 (60%)	2,414
Number of benign tumours	1,010 (40%)	761 (36%)	1,768
Unknown	316 (12%)	101 (5%)	417

Tumours diagnosed by cytology mostly originated from the mesenchym (which included all benign and malignant mesenchymal proliferations of bone and soft tissue). Second most frequent were tumours that originated from hematopoietic origin (which included histiocytomas, histiocytic sarcomas, MCT, NHL, plasma cell tumours and atypical lymphoid/histiocytic proliferations).

**Figure 1:**
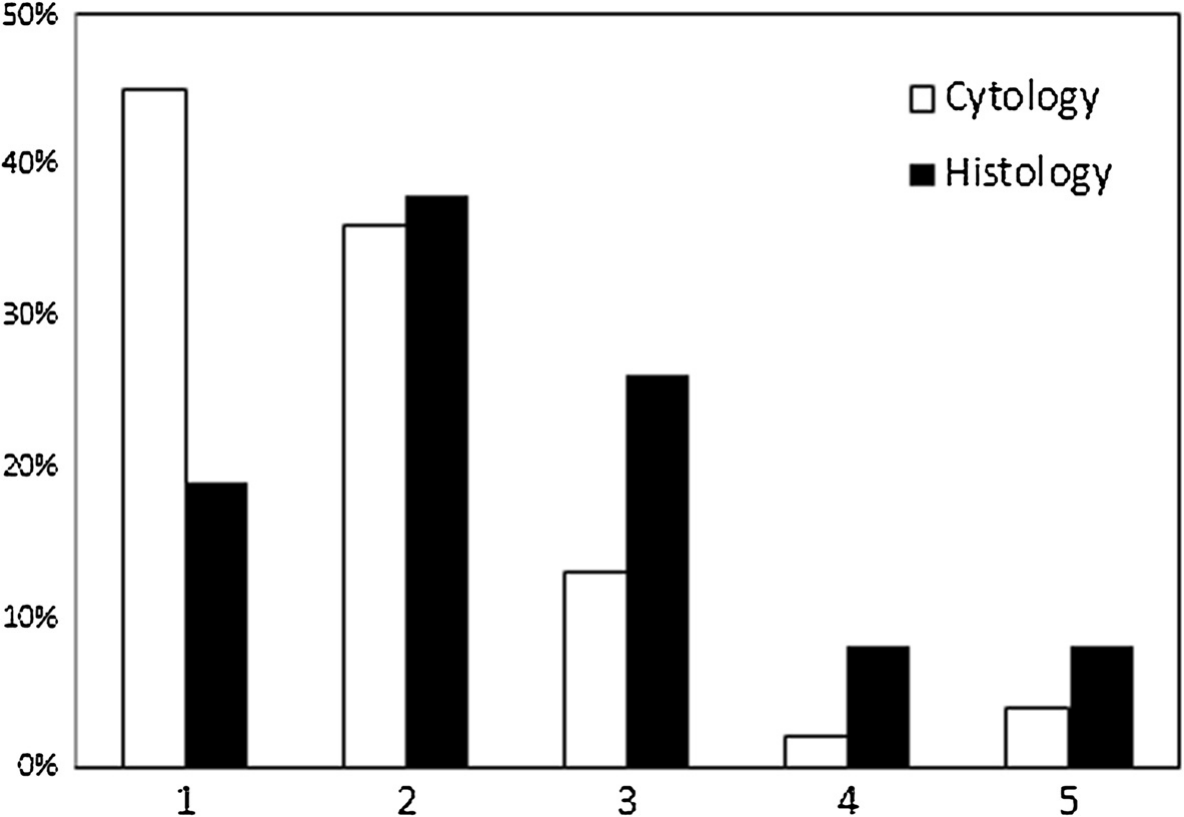
**Distribution into tissues of origin (in percentage) for the cytological- and histopathological diagnosed data-sets.** Origin: 1: Mesenchymal origin, 2: Hematopoietic/lymphoid origin, 3: Epithelial origin, 4: Neuroectodermal origin, 5: Other origins (gonadal, glial, NOS).

Tumours in the histological data-set were mostly of hematopoietic origin (which included NHL, MCT, histiocytic sarcomas, splenic nodular hyperplasias/splenomas, transmissable venereal tumours, thymomas, histiocytomas (CCH) and plasma cell tumours) followed by epithelial lesions (including benign mammary tumours, perianal gland adenomas, adenomas of other origin, ameloblastomas, basal cell tumours, epitheliomas, insulinomas, papillomas, trichoblastomas, trichoepitheliomas, and all (adeno-) carcinomas). Figure 1 shows this distribution into tissues of origin for both cytological and histological data-sets.

Both data-sets were cross-referenced for double entries and 54 cases were identified as being diagnosed with cytology as well as histology. Of these 54 cases, 18 cases were diagnosed as MCT, 16 as being a soft tissue sarcoma, six as NHL, six as carcinomas, four as peri-anal gland tumours, two as CCH, one as amelanotic melanoma and one as plasmacytoma.

The annual estimated incidence rate (EIR) was calculated, considering a population at risk of 29,304 dogs per year. Based on an average of 657 annually diagnosed tumours (using either cytology or histology), an EIR of 2,242 per 100,000 dogs was calculated for the occurrence of benign and malignant tumours in the Golden retriever dog and an EIR of 1,174 per 100,000 dogs for the development of only malignant tumours. Based merely on tumours diagnosed by histology, an EIR of 1,034 was calculated for the occurrence of all tumours- and an EIR of 615 for the development of malignant tumours, respectively (Table 2). Based on cytology alone, for the development of malignant tumours an EIR of 586 was calculated (Table 3).

**Table 2:** Estimated Incidence Rates (per 100,000 dog years at risk) of the most common types of benign and malignant histologically diagnosed tumours in the Golden retriever compared to Incidence Rates (per 100,000 dog years at risk) found in previous studies concerning the general dog population

**Histological data-set**	**EIR Utrecht**	**Incidence rate Madison, Wisconsin **[38]	**(Standardized) Incidence Rate UK **[34]	**Incidence rate Genoa, Italy **[30]	**Incidence rate Alameda, Contra Costa **[32]
General development of tumour	**1,034**		1,948	760	
Development of cancer	**615**		747.9	310	381
MCT	**265**		129		
STS	**114**	35	142		36
Melanoma	**82**	25			
CCH	**70**		377		
Benign mammary tumour	**48**		11		
Adenoma (non-mammary, non-peri-analgland	**45**				
NHL	**35**	25	114	19.9 (Males)	21.7
				22.9 (Females)	

**Table 3:** Estimated Incidence Rates (per 100,000 dog-years at risk) of the most common types of benign and malignant cytologically diagnosed tumours in the Golden retriever compared to Incidence Rates (per 100,000 dog-years at risk) found in previous studies concerning the general dog population

**Cytological data-set**	**EIR Utrecht**	**Incidence rate Madison, Wisconsin **[38]	**(Standardized) Incidence rate UK **[34]	**Incidence rate Genoa, Italy **[30]	**Incidence rate Alameda, Contra Costa **[32]
General development of tumour	**1,232**		2,671	760	
Development of cancer	**586**		748	310	381
‘Fat, suspect lipoma’	**429**		318		
MCT	**265**		129		
NHL	**121**	25	114	19.9 (Males)	21.7
				and 22.9 (Females)	
Perianal gland tumour	**68**				
(Adeno) carcinoma	**37**				
Mesenchymal proliferation, susp. STS	**37**	35	142		36
CCH	**30**		377		
Melanoma	**27**	25		0.7 (Males) 0.6 (Females)	25

**Figure 2:**
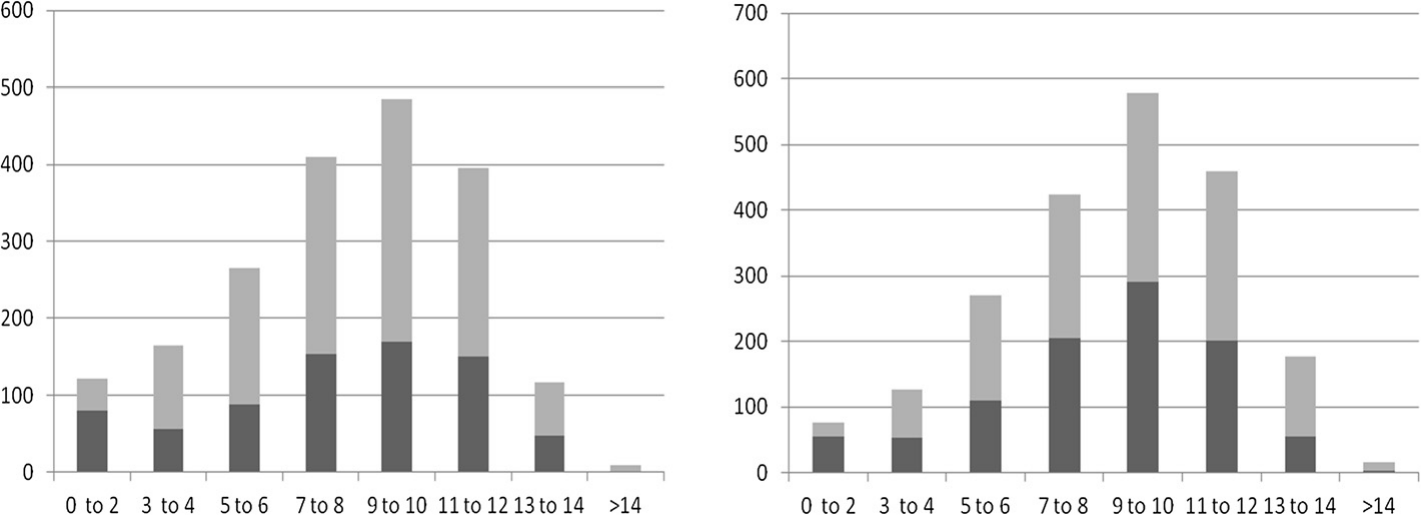
**Age distribution of benign and malignant tumours as diagnosed by means of histology (Left) or cytology (Right).** Horizontal axis: Age of the dogs (years); vertical axis: Number of cases diagnosed. Dark grey: Benign tumours. Light grey: Malignant tumours.

In general, benign tumours occurred at a younger age than malignant tumours in the tumours diagnosed using cytology (8.40 vs 8.95 yrs; Δ = 0.55 years, *P* < 0.001) as well as the tumours diagnosed using histology (7.86 vs 8.35 yrs, Δ = 0.49 years, *P* < 0.002) (Figure 2). Also, a significant difference was found in the median age of tumour-diagnosis between the two diagnostic methods (cytology: mean AOO: 8.76 yrs, histology: mean AOO: 8.19 yrs, Δ =0.57 yrs, *P* <0.001).

**Figure 3:**
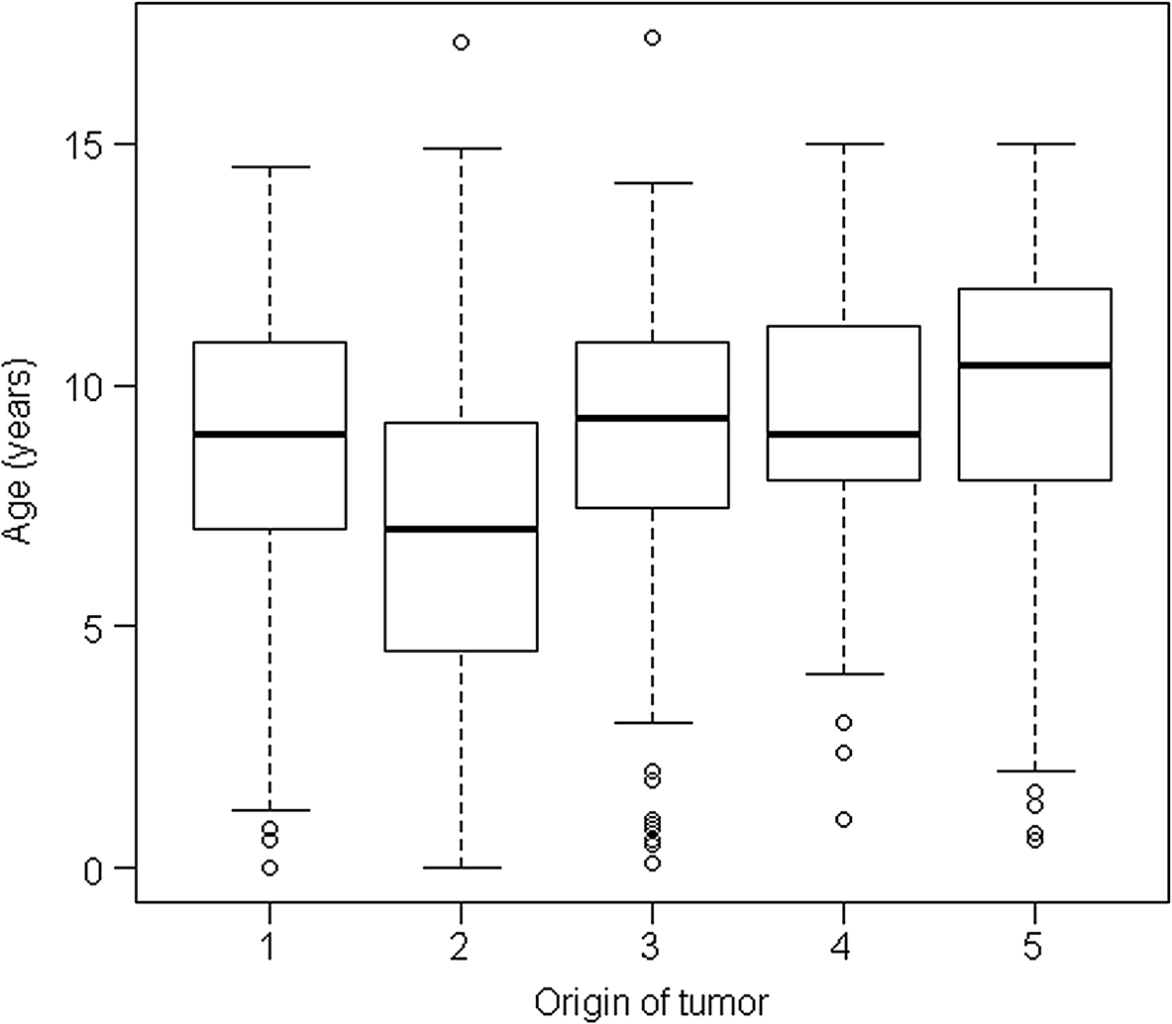
**Age-distribution in different tissues of origins in tumours diagnosed using histopathology.** Origin: 1: Mesenchymal origin; 2: hematopoietic origin; 3: Epithelial origin, 4: Neuroectodermal origin; 5: Other (‘NOS’,gonadal origin; glial tumours).

**Figure 4:**
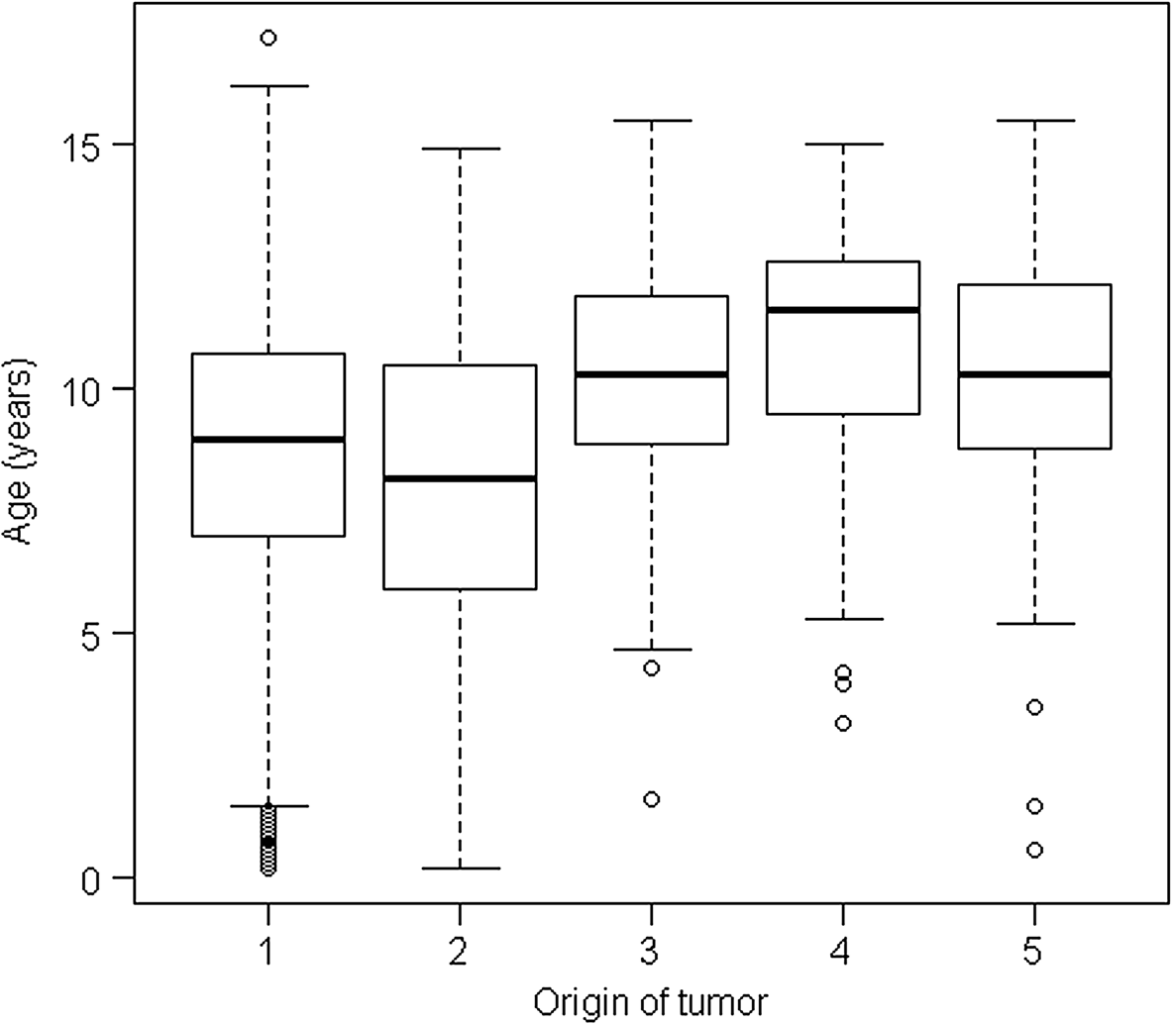
**Age-distribution in different tissues of origins in tumours diagnosed using cytology.** Origin: 1: Mesenchymal origin; 2: hematopoietic origin; 3: Epithelial origin, 4: Neuroectodermal origin; 5: Other (‘NOS’,gonadal origin; glial tumours).

In both data-sets the median age at which tumours of mesenchymal origin were diagnosed was higher (9 yrs; range: 0–17.2) than that of tumours of hematopoietic origin (8.2 yrs (range: 0.2-14.9 yrs) by use of cytology and 7.0 (range: 0–17.1 yrs) by use of histology. Figure 3 and Figure 4 show the age-distribution in all different tissues of origin.

The Male: Female ratio (M:F) of all histopathological diagnosed tumours was 1.03 and that of all cytological diagnosed tumours was 0.98. No significant difference in gender was found for tumour development. The data provided by the submitting clinic did not consistently include neutering status or date of neutering, prohibiting an examination on the potential effect of age of neutering on tumour occurrence as published recently [29].

Significantly less frequently submitted were tumours derived from internal organs (GI-tract and genital tract: 9%) compared to neoplasms that are more easy accessible (skin and adnexa, 54%).

## Discussion

### Estimated incidence rates

The percentage of malignant tumours versus benign tumours was higher in the group diagnosed by histopathology than in the group diagnosed by cytology. This difference might be caused by the relatively high percentage of cases where cytological evaluation did not allow a reliable distinction between a benign or malignant neoplasm.

A striking finding was the existence of major variation in representation for specific tumour types amongst the two data sets. This could be a result of the clinician’s expectation of how likely it will be to obtain an accurate diagnosis by either method, or the possibility that immediate removal of tissue could be therapeutic. It may however also indicate a practice in which knowledge of the tumour type by cytology decreases the likelihood that the resected mass is submitted for histopathology. Future research should examine this in more detail, since it influences the level of veterinary care and will also influence epidemiological studies. The percentage of malignant tumours as well as its EIR in the tumours diagnosed using histopathology in the current study were higher than what has been reported in other studies that considered the general dog-population; namely in Norway (2008) [18] Italy (2009) [30,31] and Alameda (1968) [32,33]. This is suggestive of a general breed-predisposition for malignant tumours in the Golden retriever. This was also the conclusion of a study from Reid-Smith et al. (Proceedings; *The incidence of neoplasia in the canine and patient populations of private veterinary practices in Southern Ontario (2000))*.

The diagnosis ‘Fat; possibly lipoma’ was clearly the most commonly diagnosed benign lesion in the cytological data-set, which was also the case in a study in Denmark [19] and the UK [34]. These last studies consider a general dog population instead of one particular breed and both include cases diagnosed by both methods and not just histology. The EIR of 429 for ‘fat; suspect lipoma’ found in the current study within our cytological diagnosed tumours, was higher than the Incidence Rate (IR) found in a study in England (IR: 317) [34]. We could however not confirm this EIR in our histolological data-set (EIR: 2.40). In a Norwegian study that included only cases diagnosed using histology [35], it was not the most common benign lesion. An explanation for this marked difference may be that not all veterinarians will have a cytologically diagnosed lipoma, or a lesion that is suspect to be a lipoma based on mere clinical examination, submitted for histopathological evaluation in our study. After exclusion of all 879 cases of ‘Fat, suspect lipoma’ from the cytological data-set, only 15% of the tumours could be considered of mesenchymal origin instead of 45%. Epithelial proliferations are then more common (20%) than mesenchymal lesions, which is consistent with our histopathological dataset as well as with previous research [34].

Of all histologically confirmed tumours, CCH was the most common benign tumour, although the EIR was lower than was expected based on previous studies [34]. The fact that the EIR in the cytological data-set is much lower than that of the histological data-set is surprising. It illustrates that CCH is diagnosed more commonly using histopathological than cytological evaluation. CCH is a benign lesion [36,37], and a study in 2007 already showed that it is easily recognizable using only cytological examination [36]. These lesions usually undergo spontaneous regression in younger dogs [36,37] making surgery unnecessary. It is our hope that CCH in future will be more commonly diagnosed by cytology, leading to a shift in the proportion diagnosed by the two methods.

In both data-sets, the most commonly observed malignant lesion is the MCT. This is consistent with other studies that evaluate the general dog-population, such as the one in Denmark [19] and Norway [18,35]. However the EIR calculated for MCT in both of the data-sets is much higher in relation to the EIR of MCT in dogs irrespective of breed than would be expected on the basis of previous reports [34]. This confirms the breed predisposition for MCT, already mentioned in earlier studies [12,20]. The same holds true for the relatively high EIR for melanomas in the histopathological data-set when compared to previous studies [21,38].

Another surprising finding was the high EIR (116) of NHL This was much higher than the IR found in most previous reports, with an IR of approximately 20 per 100,000 [30,32,38] and with a previous report by Teske et.al, that reported the IR of NHL to be at least 33 [39] for the general dog-population in The Netherlands. This higher risk of NHL in the current study might be based upon the fact that the diagnosis of NHL is usually made by cytological evaluation, a diagnostic procedure very often not included in previously reported epidemiologic studies [32,34]. This observation of an higher than average risk supports the existence of a breed-predisposition as has already been suspected [17,22,23]. The EIR found in this study is comparable to the (age-standardized) IR found recently by Dobson et al. (IR: 107) that most likely included cytological diagnoses [34].

Cytology is not very effective in further differentiating a mesenchymal lesion [40] because of morphological similarities between reactive and neoplastic fibroblasts; therefore the diagnosis ‘soft tissue sarcoma’ is usually based on histological evaluation. The EIR for STS found in our histologically diagnosed data-set (EIR: 114) is higher than some previous publications in which an IR of 35 was reported, [38] but somewhat lower than the IR found by Dobson et al. (IR: 142) [34]. This last study, however, used age-standardized IR, which we could not. A breed predisposition for STS in the Golden retriever is therefore still considered possible.

Of the STS-subtypes that could be identified by routine histopathological evaluation in 1998–2004, the most frequently found tumours were fibrosarcomas (n = 54; 23%), hemangiosarcomas (n = 34; 15%) and neurofibrosarcomas (9%), which is largely in accordance with literature [32]. However, in 40% of all STS further differentiation proved impossible, so a more precise indication of a possible predisposition for a specific subtype was impossible.

### Additional value of combining both data-sets

In this study we attempted to evaluate and explain the complementary value of combining results obtained by different diagnostic method of choice; being both cytological and histological examination. We clearly established that the incidence of NHL is underestimated in the histological data-set. Also, the difference in incidence in CCH as well as the diagnosis ‘Fat; suspect lipoma’ between the two data-sets, underlines the importance of combining both methods when performing a biopsy-based epidemiological study. The change of data registration exerted after 2004, led us to limit our survey to earlier years. Even considering a possible change in submission rates in recent years, the low number of patients (1.8%) that were sent in for both cytological diagnosis as well as for histological diagnosis was still surprising, in particular with respect to the importance of achieving an assessment of grade and completeness of excision by histopathology after getting a diagnosis of tumour type by cytology. Too often – it appears - do veterinarians opt for either cytology or histology for diagnostic purpose. A growing awareness of the strength of combining both cytology and histology, can perhaps in future change this observation.

Also based on our results we therefore believe that optimal veterinary care of dogs suspected of neoplasia is best exerted by presurgical cytological examination of FNABs followed by histopathology of resected masses or, in cases deemed not manageable by surgery, by histopathology of tissues biopsies if cytology was inconclusive. Furthermore, such practice serves epidemiological studies of neoplasia.

### Age, sex and location

As is shown in Figure 1, the incidence for development of a neoplasm was relatively low in younger animals, increased sharply after the age of three, and peaked at 9 years for the histopathological- and at 10 years for the cytological data-set. An age-dependent increase in incidence for the development of a neoplasm is in agreement with other studies [8,31,34]. Highest-peak-incidence was noted at a younger age in the current study than peak-incidence of cancers in a study in Italy (>12 years) [31] but at a comparable age when compared with the study of Dobson [34], which is remarkable, considering the potential bias for younger animals of this last study due to different age-structure [23]. Also, as was the case in a general dog-population studied in Denmark [19], benign lesions occur at a younger age than malignant lesions in both data-sets.

There is a significant difference (*P* < 0.001) in mean age of animals when comparing the two data-sets. Even when excluding CCH, a tumour reported to occur at a young age [41] as was confirmed in our study (median age 2.8 years and 2.9 years, respectively, for the cytologically- and histopathologically diagnosed tumours), this difference remained significant (*P* < 0.001). In accordance with literature [19], there appeared no sex predisposition for general tumour development. The data provided by the submitting clinic did not consistently included neutering status or date of neutering, prohibiting an examination on the potential effect of age of neutering on tumour occurrence as published recently [29].

Taking a potential sampling bias for the occurrence of tumours into account, since more common locations are also more easily clinically accessible [8], the most common locations of histological diagnosed tumours were skin and adnexa (Table 4) which was also found in earlier studies [12,17].

**Table 4:** Distribution of location of 2,124 tumours diagnosed using histopathology

**Location**	**Frequency**	**Percentage**
Head (excluding skin/adnexa)	308	15
Skin and adnexa	1148	54
Mammae	187	9
Gastro-intestinal tract	72	3
Endocrine organs	12	0.6
Genital tract	118	6
Hematoproliferative system	58	3
Urogenital tract	12	0.6
Heart and lungs	6	0.3
Central nervous system	6	0.3
Soft tissue, other	143	7
Other	54	3

‘*Head*’ includes eye, nose mouth and sinus; ‘*skin and adnexa*’ includes skin and adnexa head, skin and adnexa (other), salivary gland, perianal gland, analsac, axial, abaxial, extra-skeletal; ‘*gastro-intestinal tract*’ includes stomach, pancreas, liver, bile duct, intestines; ‘*endocrine organs*’ includes adrenal gland, thyroid- and parathyroid gland; ‘*genital tract*’ includes male and female reproduction organs, ‘*hematoproliferative system*’ includes spleen, liver, thymus, lymph nodes.

Veterinary cancer registries are few in number and scattered [32,42]. Also, there is little information on age, incidence, type, location and behavior of tumours in canine populations in general [32,42]. A population-based cancer registry is preferred over a hospital-based cancer registry, because it aims to represent all cases in a known population [43], whereas hospital-based cancer registries such as the ones in America [44,45] and Italy [46] do not include cases that were seen only by primary care veterinarians, risking a potential bias [47].

Much of what we know so far on tumour incidence derives from the population-based veterinary cancer registry in California, the California Animal Cancer Registry (CANR) [32]. Results from this study are frequently used as reference data set but are more than 40 years old and were obtained in one specific region only.

More recently, various studies came to rely on insurance data, e.g. such as the ones in England [23,34] and Sweden [48,49]. This kind of research could lead to a potential age-related bias, since older dogs are less often insured [34,50] and also excludes a presumed high portion of dogs that are not insured at all [47]. Additionally, diagnostic validation by histopathological or cytological examination, in addition to a diagnosis based upon clinical manifestation, is in some instances lacking. This may lead to uncertainty regarding the accuracy of the recorded diagnosis [43]. As an alternative method, some researchers have chosen to rely on veterinary cancer registries, like the study in Denmark [19], or questionnaires, such as studies in Norway [51] and Denmark [16]. These approaches carry the risk of a voluntary bias, because it is unlikely that owners report all tumours to the registry [16,43]. Also, regarding surveys, the overall response rate is expected to be only half the sample population and includes a potential bias in responders versus non-responders [51]. The Norwegian Canine Cancer Register [52] and a study in Italy [31] tried to improve the number of diagnosed cases by offering free of charge histopathological examination of all tumours of dogs in four counties. However, cytological examinations were not included in this study, and studies that offer free of charge will remain an exception due to financial and logistical challenges.

In this study, we chose to assess the incidence of tumours in one specific dog breed, with a centrally accessible source of diagnostic data using a broad system of tumour classification. As was the case in other studies [34], also in our study there was risk of a potential bias in both numerator and denominator. In our study, the strongest bias is most likely caused by the fact that not all private clinicians have their biopsies evaluated at the Utrecht University of Veterinary Medicine (UUVM). Note that the two participating laboratories are not the only diagnostic laboratories in the Netherlands. As samples numbers analysed by commercial laboratories are confidential we can only make a best estimated guess that some 20-40% and 40-50% of all histologically or cytologically diagnosed tumours were evaluated, respectively. This certainly will cause an underestimation of the true incidence. On the other hand a potential overestimation exists in the fact that the reference population is composed of pedigree-dogs as registered at the Dutch Kennel club, while biopsies in some cases derive from dogs that are registered by owners as ‘Golden retrievers’ but that lack a pedigree.

In addition, an unknown portion of tumours remain undiagnosed. Reason for this, is the fact that not all dogs are presented to a veterinarian and not all owners are willing to pursue and pay for a diagnostic work-up [52]. This situation is perhaps even more likely, if the population at risk is not insured like the one used in this study. We therefore consider the incidence rates found within this study to be an estimation of the true incidence rate. Most other studies take note of encountered bias, but continue to register incidence rates (IR). Because of expected variations in both numerators and denominator, it proved difficult to compare this EIR with IR found in previous research [30,32,34,38,53,54]. Our EIR is lower than the IR for tumour development in the general dog population found in a Canadian study (IR: 3,965) but this study makes use of a computerized medical record system instead of only histopathological and cytological data (Proceedings; *The incidence of neoplasia in the canine and patient populations of private veterinary practices in Southern Ontario* (2000) and therefore is likely to have a very different denominator.

More comparable are the IR calculated for tumour development in the general dog population (not breed-specific) found by the CANR (IR:1,134) [30], a study in England (IR: 1.948) [34], and a study in Italy (IR: 282) [31]. The high EIR found in the present study when both of the data-sets are combined (EIR: 2,242), could be an indication of a breed-predisposition for general tumour development in the Golden retriever.

## Conclusions

The high EIR found in this study when evaluating comparable research is an indication of a breed-predisposition for cancer as well as general tumour development in the Golden retriever. The breed predisposition for MCT, NHL and melanoma in Golden retrievers was confirmed. There are also indications for a predisposition for STS.

Comparable to previous research that considered the dog-population in general, benign lesions occur at a younger age than malignant tumours, and most tumours develop in the skin. There appears no gender predilection. Including diagnoses made through histopathology as well as cytology, reduces the risk of a bias based on the diagnostic procedure of choice. A study combining both diagnostic procedures is therefore of greater value than a study that focuses on a single diagnostic procedure.

## Method

The experimental protocol (ID 2007.III.08.110) was peer-reviewed by the scientific committee of the Department of Animals in Science & Society, Utrecht University, The Netherlands, and approved by the Animal Experiments Committee of the Academic Biomedical Centre, Utrecht, The Netherlands. The Animal Experiments Committee based its decision on ‘De Wet op de Dierproeven’ (The Dutch ‘Experiments on Animals Act’, 1996) and on the ‘Dierproevenbesluit’ (the Dutch ‘animal experiments decree’, 1996). Both documents are available online at http://wetten.overheid.nl.

In this retrospective study, two separate data-sets were used, consisting of either cytologically or histopathologically confirmed tumours from the client-owned pet-population of Dutch Golden retrievers that were submitted during the period 1998–2004 for cytological examination to Utrecht University Veterinary Diagnostic Laboratory (UVDL), or for histological examination during that period to the *Veterinary Pathologic Diagnostic Centr*e (VPDC). Permission to use these data sets was obtained from the Departments of the Veterinary Faculty concerned. As in previous research [32] dog breeds were recorded as stated by the owner. The material was obtained from patients seen within the UUVM as well as from primary clinics, referral hospitals and private practitioners from all over the Netherlands.

In incidental cases, when detailed information was unavailable, variables selected for investigation were age, sex and, in the histopathological data-set, site of biopsy. If in an animal multiple tumours were detected during the period of the study, these were recorded as separate incidences. A broad system of tumour classification was applied for both data sets, which was based on tissue of origin and actual diagnosis. All tumours were divided into six (for the cytological data-set) or seven (for the histopathological data-set) tissues of origin (mesenchymal, hematopoietic, epithelial, neuroectodermal, gonadal plus for the cytological tumours: malignant Not Otherwise Specified (NOS) and for the histopathological data-set: glial tumours and tumours that could be NOS).

### Numerator and denominator

In total, 18,648 Golden retrievers (9,639 male dogs and 9,009 bitches) were registered between 1998 and 2004 in the Raad van Beheer (Dutch Kennel Club), the principal cynological organization in the Netherlands. This resulted in an average entry of 2,664 animals per year. The Golden retriever reaches an average age of 11 years (http://en.wikipedia.org/wiki/Golden_Retriever), so a cross-sectional estimation of the total population during one year is expected to be 11 * 2,664 = 29,304 (15,147 male dogs and 14,157 bitches). This was defined denominator, or population at risk (D).

The annual Estimated Incidence Rate (EIR) was calculated as the observed number of cases (C) in one year, calculated for a population that consists of 100,000 individuals.

$$\text{EIR}=\left(\frac{C}{\text{7 yrs}}\right)\times \left(\frac{100,000}{D}\right)$$

Input was considered as two separate data sets, and thus two separate numerators were all neoplastic biopsies of dogs registered as ‘Golden retriever’ that were sent during the given period to either the VPDC or the UVDL. Results were used to calculate the occurrence of neoplastic types in the two data sets and by calculation of the number per annum corrected for the share of the two laboratories of all submissions – to exclude double counts - in the entire (with or without pedigree) Dutch Golden Retriever population to assess an EIR.

### Statistics

A Student’s t-test was used to test the age difference between malignant and benign tumours, a *P* < 0.05 was considered to be significant. Normality and constancy of variance of the data was evaluated by inspecting the histograms. Statistical analyses were performed in R library version 1.7 (http://cran.r-project.org).

## Competing interests

The authors declare that they have no competing interests nor did they have any competing financial interests in relation to the work described.

## Authors’ contributions

KB participated in the conceptualization and design of the study (including the statistical analyses) and drafted the manuscript. ET conducted the cytology review, participated in the statistical analyses and assisted in drafting the manuscript. GG conducted the histopathology review and assisted in drafting the manuscript. LoB and LiB participated in the data-base record review. GR conceptualized and designed the study, and participated in its coordination and helped to draft the manuscript. All authors read and approved the final manuscript.
